# Time-Variant Genetic Effects as a Cause for Preterm Birth: Insights from a Population of Maternal Cousins in Sweden

**DOI:** 10.1534/g3.116.038612

**Published:** 2017-02-28

**Authors:** Julius Juodakis, Jonas Bacelis, Ge Zhang, Louis J. Muglia, Bo Jacobsson

**Affiliations:** * Department of Obstetrics and Gynecology, Institute of Clinical Sciences, Sahlgrenska Academy, University of Gothenburg, 416 85, Sweden; † Department of Obstetrics and Gynecology, Sahlgrenska University Hospital Östra, 416 85 Gothenburg, Sweden; ‡ Human Genetics Division, Cincinnati Children’s Hospital Medical Center, Ohio 45229; § Center for Prevention of Preterm Birth, Perinatal Institute, Cincinnati Children’s Hospital Medical Center, Ohio 45229; ** March of Dimes Prematurity Research Center Ohio Collaborative, Cincinnati, Ohio 45229; †† Department of Genetics and Bioinformatics, Area of Health Data and Digitalisation, Norwegian Institute of Public Health, 0473 Oslo, Norway

**Keywords:** survival analysis, Cox regression, genetic simulations, nonproportional hazards

## Abstract

Preterm delivery (PTD) is the leading cause of neonatal mortality worldwide, yet its etiology remains largely unexplained. We propose that the genetic factors controlling this trait could act in a nonuniform manner during pregnancy, with each factor having a unique “window of sensitivity.” We test this hypothesis by modeling the distribution of gestational ages (GAs) observed in maternal cousins from the Swedish Medical Birth Register (MBR) (*n* = 35,541 pairs). The models were built using a time-to-event framework, with simulated genetic factors that increase the hazard of birth either uniformly across the pregnancy (constant effect) or only in particular windows (varying effect). By including various combinations of these factors, we obtained four models that were then optimized and compared. Best fit to the clinical data was observed when most of the factors had time-variant effects, independently of the number of loci simulated. Finally, power simulations were performed to assess the ability to discover varying-effect loci by usual methods for genome-wide association testing. We believe that the tools and concepts presented here should prove useful for the design of future studies of PTD and provide new insights into the genetic architecture determining human GA.

PTD is a major burden on public health worldwide, defined by the World Health Organization as birth before 37 completed weeks of gestation. It is the leading cause of neonatal mortality in the world, with an estimated 14% (1.1 million as of 2010) of deaths in newborns attributed to this syndrome (Liu *et al.* 2012). In addition, surviving preterm-born children are at increased risk for a wide range of adverse outcomes, ranging from a subtle decrease in school performance to severe neurological disabilities (Mwaniki *et al.* 2012; Chan *et al.* 2016). Despite such impacts and the amount of resources devoted to the problem, neither prevention nor prediction of PTD has had major success so far.

It is known that the tendency to deliver preterm is, in part, heritable. A handful of studies have analyzed the heritability of GA in large cohorts, estimating the broad-sense genetic effects to be between 25 and 50% (Clausson *et al.* 2000; Treloar *et al.* 2000; Lunde *et al.* 2007; York *et al.* 2009, 2010, 2013; Boyd *et al.* 2009; Svensson *et al.* 2009; Wu *et al.* 2015). However, such analysis is particularly complicated because of the interplay between the maternal and fetal genomes, and estimates of their relative contributions vary widely. Both maternal and fetal genomes have been investigated in genome-wide association studies (GWAS) of PTD, but no significant hits were obtained (Uzun *et al.* 2013; Zhang *et al.* 2015; Bacelis *et al.* 2016). While some candidate gene studies reported significant discoveries, these results generally fail replication attempts. Usual suspects in these studies include genes encoding matrix metalloproteinases, the progesterone receptor, and various cytokines (Bezold *et al.* 2013; Geng *et al.* 2016).

An obvious but often overlooked fact in medical epidemiology is that GA is indeed a time-to-event variable. As such, it lends itself well to time-to-event statistical models, also referred to as survival analysis [an overview is presented in many statistical textbooks and reviews, such as Zwiener *et al.* (2011)]. In the simplest formulation, survival models consist of predictors that multiply the baseline hazard of an event by a constant factor across the entire period of observation. This approach has been utilized in several epidemiologic and two genetic studies of PTD (Hatch *et al.* 1998; Smith 2001; Hernandez *et al.* 2010; Harper *et al.* 2011; Auger *et al.* 2014; Park *et al.* 2014; Mitchell *et al.* 2015).

However, it is likely an oversimplification to assume that factors influencing neonatal outcomes act uniformly across the duration of pregnancy. Indeed, “windows of sensitivity”—periods when a certain structure or trait is particularly affected by a given exposure—are a well-known concept in developmental toxicology (Kapp and Tyl 2010). The possibility of time-varying effects in PTD epidemiology has been explored in several studies concerning exposure to particulate matter in air (Chang *et al.* 2012, 2015; DeFranco *et al.* 2016; Nachman *et al.* 2016) or phthalates (Adibi *et al.* 2009; Meeker *et al.* 2009; Huang *et al.* 2014; Ferguson *et al.* 2014a,b). Ferguson *et al.* (2014a) also proposed that different subtypes of PTD could have different windows of sensitivity; in their study, spontaneous PTD was mostly correlated to third trimester phthalate exposure, while “placental” PTD was most sensitive to phthalate exposure early in pregnancy. Undoubtedly, identifying further differences in susceptibility timing could lead to insights about the mechanisms controlling human pregnancy and PTD.

In this study, we propose that genetic factors could influence the risk of PTD in a time-dependent manner. Building on the framework of heritability studies, we obtain a conditional distribution of GAs in related individuals from a Swedish population register. This distribution can be modeled as a time-to-event trait, determined by simulated susceptibility loci. We proceed to test our hypothesis by comparing the fit of models with and without time-varying genetic effects.

## Methods

### Correlation patterns in the Swedish MBR

Clinical data for this study was obtained from the Swedish MBR, collected on all deliveries nationwide. We included all singleton live births with spontaneous onset of delivery from 1992 to 2012. Phenotype of interest (GA) was obtained by ultrasound measurement in >90% of pregnancies. To account for known environmental covariates, a multivariable regression model was fitted to this sample, using known maternal and fetal predictors of GA (listed in Supplemental Material, Table S1 in File S1). At this stage, we use basic linear regression to avoid any assumptions about time dependency of the covariate effects. In total, the study population comprised 1,384,130 deliveries with full information on all predictors. All further analyses use residuals obtained from this model (added to the population mean) as the adjusted GA.

We then proceeded to identify all pairs of full nontwin sisters whose pregnancies were included in our study population. For mothers who delivered more than once, we retained only the pregnancy of lowest parity available in the data, resulting in 35,541 pairs of individuals. To inspect patterns of correlation in their pregnancies (= GA of cousins), we plotted the conditional distribution of the first cousin’s phenotype in each pair, given the phenotype of the second cousin in that pair (cousins assigned to “first” and “second” randomly). Furthermore, to quantify this distribution, GA_2_ was binned into 7-d windows, and 5th, 25th, 50th, 75th, and 95th percentiles of GA_1_ were calculated for each bin. For example, the 50th percentile of the 280-d bin can be interpreted as the median pregnancy length for a woman whose sister delivered between 280 and 286 d (after adjusting for environmental factors). Only bins with >100 individuals were retained and used for model fitting. For comparison purposes, height correlation patterns were obtained from pairs of sisters in the same way, except that 5-cm windows were used for binning and no adjustment for environmental factors was made.

### Simulation models

#### Baseline model structure:

We built simulation models in a survival analysis framework, designed to investigate how introduction of various types of genetic risk factors could affect the correlation patterns in related individuals.

Initially, we selected the most appropriate baseline hazard function based on overall fit to the Swedish MBR using R package “flexsurv,” version 0.7.1 (Jackson 2016). Three models were tested: exponential, Weibull, and Gompertz. At this stage, all >1.3 million regression-adjusted GAs were used, excluding the previously identified set of cousins. We consider GA < 150 d as unviable, hence day 150 was set as the time when pregnancies enter the population “at risk” for a live birth (*i.e.*, day 150 corresponds to *t* = 0 and day 151 to *t* = 1 and so on). Based on the lowest AIC value, Gompertz function was chosen for further model building, with the baseline hazard function (corresponding to the instantaneous rate of births) $\lambda_{0}\left(t\right)=\lambda e^{\alpha t}.$ Basic dynamics of such a model are presented in Figure S1 in File S1. We continue to use the optimal λ (rate) and α (shape) values obtained at this stage in all further simulations.

#### Simulation of genetic risk factors:

Conceptually, genetic effects are entered into the model as simulated diploid diallelic loci of the mother’s genome, with the minor allele conferring either susceptibility to or protection from PTD. The two allele copies in each locus are assumed to act independently of each other and of other loci (linkage disequilibrium is not modeled). In order to explore the effects caused by higher numbers of susceptibility loci, while at the same time maintaining a limited number of parameters to allow an exhaustive search, we constrained each model to a predetermined number of different classes of loci. The parameters for loci within each class are identical (*i.e.*, when *n* = 3, all three loci have equal effect size). In constant-effect classes, three parameters can be varied: minor allele frequency (MAF) *p*, number of loci *n*, and effect size γ. These loci act as classical time-invariant covariates, *i.e.*, increase the hazard function proportionally across the entire range of GA. However, we emphasize that γ does not correspond to the common meaning of effect size in genetics and cannot be directly translated into a shift of mean trait value. In varying-effect classes, five parameters can be varied: the three parameters for the constant-effect classes and also time of peak effect μ and spread of effect σ (both measured in days of gestation).

The genetic effect (*E*_i_) contributed by one varying-effect allele at locus *i* and time *t* is defined using a Gaussian function:

$$E_{i}\left(t\right)=\frac{\gamma_{i}}{\sigma \sqrt{2\pi }}e^{-\frac{1}{2}{\left(\frac{t-\mu }{\sigma }\right)}^{2}}\;$$

This function was chosen as a simple way to generate smoothly appearing and disappearing effects, and to allow intuitive explanation of the peak effect and spread parameters. Effects for constant-effect loci are simply $E_{i}\left(t\right)=\gamma_{i}.$ Overall, the hazard function implementing shared genetic (from *N* total loci) and unique environmental contributions is:

$$\lambda \left(t\right)=\lambda e^{\alpha t}e^{\sum_{i\in N}G_{i}E_{i}\left(t\right)}$$

Here, *G_i_* is the individual’s genotype at this locus (0/1/2), and other parameters are as described above.

As we are interested in the maternal genetic effects determining GA of cousins, or, equivalently, pregnancy length of full sisters, we simulate the *N* genotypes for pairs of full siblings. Genotypes are drawn randomly assuming Hardy–Weinberg equilibrium. For instance, the probability for two siblings to have zero and two copies of the minor allele requires two heterozygous parents (frequency *2pq*), and the production of desired homozygotes at each cross (frequency 1/4 for each sibling). The product 2pq*2pq*1/4*1/*4* is then the joint probability of obtaining such a pair of siblings. The full joint distribution used to generate correlated draws is presented in Table 1.

**Table 1:** Joint probabilities for simulating genotypes of sibling pairs

Genotype of Sibling 1	*p*(*Sibling* 2 *=* 0)	*p*(*Sibling* 2 = 1)	*p*(*Sibling* 2 = 2)
0	*q*^2^(*q* + *p*^2^/4)	*pq*^2^(1 − *p*/2)	(*pq*/2)^2^
1	*pq*^2^(1 − *p*/2)	*pq*(1 + *pq*)	*p*^2^*q*(1 − *q*/2)
2	(*pq*/2)^2^	*p*^2^*q*(1 − *q*/2)	*p*^2^(*p* + *q*^2^/4)

Frequency of the minor allele is set by the parameter *p*, then *q* = 1 − *p*.

#### Implementation of the models:

In total, five models were constructed, presented in Table 2. In models consisting of constant-effect risk factors, analytical expressions were used to generate survival times, as described in File S1. In models consisting of varying-effect risk factors or a mixture of constant- and varying-effect factors, survival times were generated iteratively. In that case, the genotypes, effect sizes, and baseline parameters were used to calculate the hazard for each individual at each day of gestation, starting at day 150. This hazard was then compared against U ∼ Unif(0,1), and events (births) were assigned when $e^{-\lambda (t)}>U.$ R code used for this process is available online at https://github.com/PerinatalLab/SE_MFR_FAMILIES/blob/master/simulate_time-to-event_clean.R. To ensure that different computer implementation was not contributing to observed differences between models, final evaluation of 20 best parameter combinations for each model was done using the iterative simulator. Additionally, 20 best parameter combinations for model M2 were analyzed in 20 replications with each simulator, in order to evaluate differences between the resulting costs. All simulations and analyses were implemented in R (version 3.3.0) and C++ (R integration provided by packages “Rcpp” and “RcppArmadillo”).

**Table 2:** Details on the five models constructed in this study

Model	Constraints	Optimized Inputs	MM	ED	Notes
M0	—	—	No	0	No genetic factors
M1	γ*pn*	γ, *p*, *n*	Yes	2	One constant-effect factor
M2	∑γpn; *n*_2_ = 1	γ_1_, *p*_1_, *n*_1,_ γ_2_, *p*_2_	Yes	4	Two constant-effect factors
M3	∑γpn; *n*_2…5_ *=* 1; σ_2…5_ *=* 10; μ_2…5_ *=* {230, 237, 244, 258}; γ_2…5_ *=* {100, 80, 60, −100}; *p*_2…5_ *=* {0.005, 0.01, 0.015, 0.3}	γ_1_, *p*_1_, *n*_1_	Yes	2	One constant- and four varying-effect factors
M4	γ_1_, *p*_1_, *n*_1_ from best fit of M3; *p*_2…5_ and *n*_2…5_ as in M3	γ_2…5_	No	4	Five constant-effect factors, fixed *p* and *n*

“ED” is the equivalent number of free input parameters, taking into account the constraints and metamodeling. “MM” indicates whether MM was used to fit the model. MM, metamodeling; ED, effective dimensionality.

#### Parameter estimation:

Best-fit parameter combinations were identified using meta-modeling, followed by exhaustive random search. Input parameters for the constant-effect factors were generated by independently drawing γ∼Unif(−5, 5),p∼Unif(0, 0.5),n∼Pois(1)+1 for each factor class. To evaluate the resulting fit, sum of squares was chosen as the cost function, equal to squared distances between simulated and observed values of five quantiles (5th, 25th, 50th, 75th, and 95th percentiles) at each bin of GA. Based on the SE of sample quantiles (Cheung and Lee 2005), the sum of squares for each bin was also weighted by the square root of the number of individuals in the bin.

We noticed that$\;\sum_{i\in N}\gamma_{i}p_{i}n_{i}$ (further denoted ∑γpn) is a strong predictor of the overall cost, hence we used a meta-modeling approach; initially, a narrow interval of ∑γpn associated with the smallest costs is determined, then parameter space is constrained with these ∑γpn values and searched exhaustively. More details on the parameter estimation are presented in File S1.

#### Calculating power and type I error rate:

Power and type I error rate were calculated empirically, using simulated data from model M3. First, a sample of genotypes and corresponding phenotypes for 100–50,000 individuals was generated using the best-fit parameters for this model. Genotypes were encoded assuming additive effects (0/1/2). Besides the causal loci, two control loci with MAFs 0.015 and 0.3, but no effect, were included to calculate the type I error rate. Linear and Cox regression models were then applied to test each locus. In the former model, the univariate linear regression p-value was reported. Survival test p-values represent Wald statistics, generated by fitting a Cox model with one locus as a covariate using R package “survival.” The entire procedure was repeated 5000 times with each sample size. The fraction of p-values generated for each causal locus that were below the genome-wide significance threshold of 5 × 10^−8^ was reported as the power for the given sample size.

### Data availability

Swedish MBR data used in this study is available upon request from the National Board of Health and Welfare (Socialstyrelsen; http://www.socialstyrelsen.se/register/halsodataregister/medicinskafodelseregistret). R code developed for the analyses is available in a public repository (https://github.com/PerinatalLab/SE_MFR_FAMILIES/) and upon request.

## Results

### GA of maternal cousins shows a nonuniform correlation pattern

**Figure 1:**
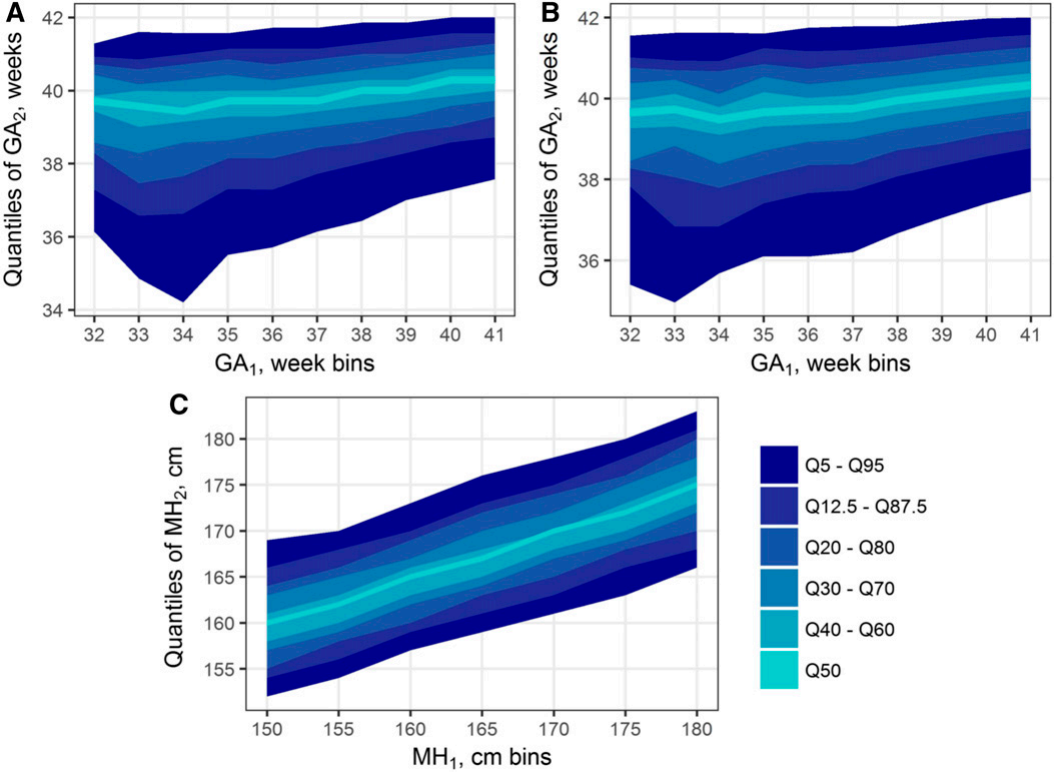
Correlation patterns observed in maternal cousin pairs in the Swedish Medical Birth Register. (A and B) Key quantiles of GA of cousin 2, conditioned on GA bin of cousin 1 [(A) unadjusted for known covariates and (B) adjusted]. (C) Quantiles of maternal height of cousin 2, conditioned on maternal height bin of cousin 1 (*i.e.*, distribution of sisters’ heights). Cousin assignments within each pair (one or two) arbitrary. While height quantiles ascend uniformly across the *x*-axis bins, it is not the case for gestational age, even after adjustment for known environmental effects. GA, gestational age; GA_1_, GA of cousin 1; GA_2_, GA of cousin 2; MH, maternal height; MH_1_; MH of cousin 1; MH_2_, MH of cousin 2.

Using information present in the Swedish MBR, we obtained a conditional distribution of GA in maternal cousins, showing a clear and unique pattern of correlation (Figure 1A). The bottom quantiles for GA have a steeper slope than the median or top quantiles: as the GA of the first cousin in each pair increases from 33 to 41 wk, the 95th percentile of second cousin’s GA climbs by 5 d, while that of the 5th percentile increases by almost three wk. This pattern persists almost unchanged after adjustment for known environmental covariates (Figure 1B). Full details of the regression model used to obtain GA residuals are presented in Table S1 in File S1. In contrast, maternal height, obtained from the same cousin pairs (*i.e.*, sisters’ height) does not show such a pattern (Figure 1C). All height quantiles ascend at approximately similar slope, without distortions even at the extremes of the range, where bins contain much fewer individuals.

### Establishing a model with time-invariant genetic effects

**Figure 2:**
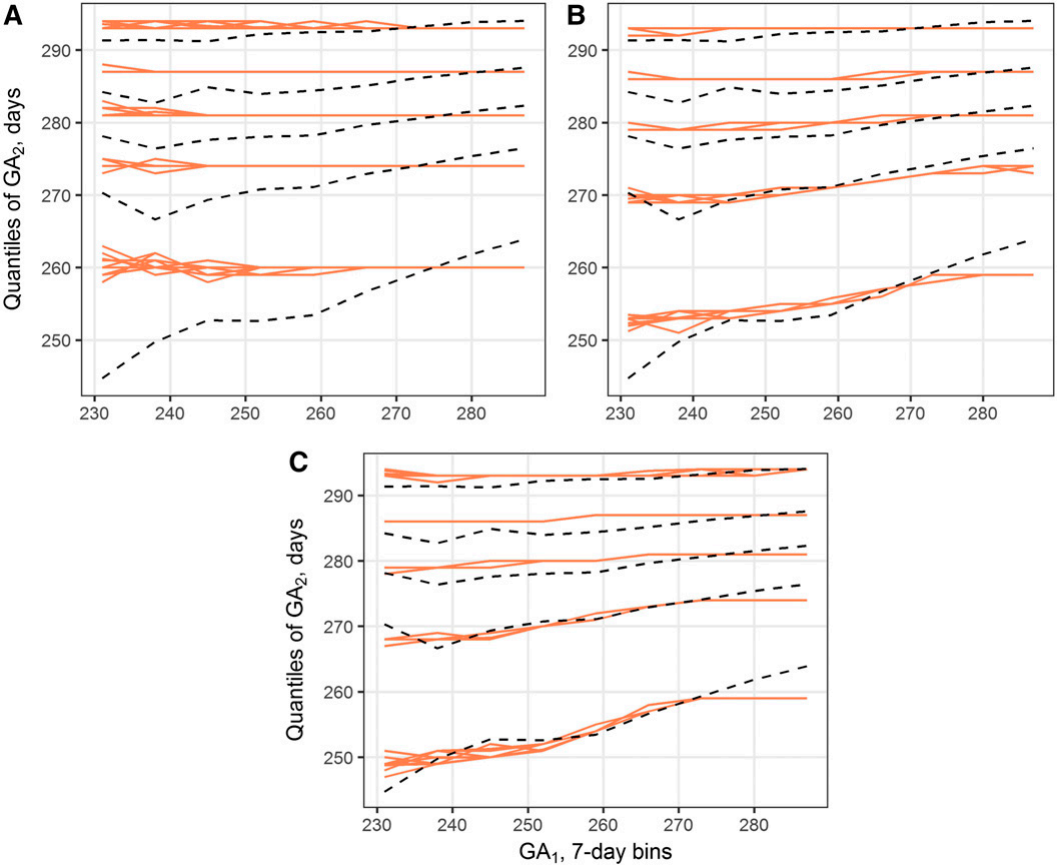
Best-fit simulation results for models M0 (A), M1 (B), and M2 (C). Red lines show simulated 5th, 25th, 50th, 75th, and 95th percentiles of GA_2_, conditioned on GA_1_ bin. Results from 10 iterations are overlapped. Black dashed lines are the corresponding quantiles observed in Swedish MBR. GA_1_, gestational age of cousin 1; GA_2_, gestational age of cousin 2.

To test whether the observed GA correlation patterns could be explained by shared genetic factors, we have built and explored a stochastic model of *in utero* survival time in cousins. We started by comparing models M0, containing no genetic effects, and M1, containing one class of loci with time-invariant effects on the hazard of birth. Mean sum-of-squares associated with model M0 was 1829 (SD 58.6), and the resulting fit is shown in Figure 2A. As expected, phenotypes created in this way show no correlation among relatives.

Clearly, model M1 fits the observed data better than M0, with the lowest observed sum-of-squares of 796 (SD 51.2). Ten input combinations resulting in the lowest costs are presented in Table S3 in File S1 (it should also be noted that all of the best combinations correspond to one or more rare mutations with high effect sizes, rather than common variants with smaller effects.) However, even though the best fit of this model has less than half the cost of M0, model M1 does not fully reproduce the quantile pattern observed in the register data, as can be seen in Figure 2B. The strongest deviations between simulated and observed quantiles are seen in the lowest and highest GA_1_ bins.

Model M2 was used to test whether introducing more classes of genetic factors could have an effect on the fit. This model contains one class of loci with three parameters, as in model M1, and an additional class with two parameters, *γ*_2_ and *p*_2_ (*n*_2_ is set to 1). The 10 best input combinations for this model are presented in Table S4 in File S1. Remarkably, almost all of the best combinations have an effect size between 2.4 and 2.8 for one class, and a small negative effect for the other class. To put this in context, a γ in the interval (2.4; 2.8) is observed in <7% tested inputs. Compared to model M1, addition of parameters led to some improvement in the best-fit cost (from 796 to 655, SD 19.9), but only minimal change in overall quantile patterns (Figure 2C).

### Introduction of time-variant effects leads to a marked improvement in model fit

**Figure 3:**
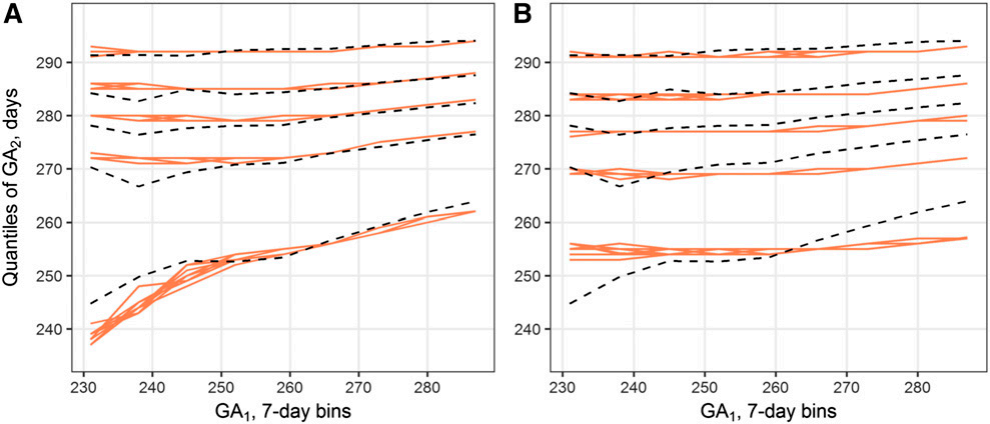
Best-fit simulation results for models M3 (A) and M4 (B). Red lines show simulated 5th, 25th, 50th, 75th, and 95th percentiles of GA_2_, conditioned on GA_1_ bin. Results from 10 iterations are overlapped. Black dashed lines are the corresponding quantiles observed in the Swedish Medical Birth Register. GA_1_, gestational age of cousin 1; GA_2_, gestational age of cousin 2.

We then proceeded to test our hypothesis that the observed correlation patterns could be explained by genetic factors with time-variant effects. Besides the parameters used for constant-effect loci, classes of time-variant factors in our model require two additional inputs (time of the largest effect and size of the sensitivity window). Given >1 such class, an exhaustive search of the parameter space becomes infeasible. Hence, for time-variant factors we used predefined inputs that were selected based on responses observed in simpler models, and estimated only the parameters for constant-effect loci. Model M3 contains one class of the latter type and four classes of varying-effect genetic factors. Ten best combinations of the estimated inputs are presented in Table S5 in File S1. Optimal fit found in this model has a much lower cost than in previous models (338 *vs.* 655, SD 30.0); furthermore, the simulated quantiles match the observed patterns throughout all GA_1_ bins up from ∼250 d (Figure 3A). As the parameters for varying-effect loci were selected manually, we performed sensitivity analysis to investigate whether small perturbations in the parameter values could lead to significantly different results (File S1). The model appears to be stable across the tested range of *p* and γ values, although shift in timing, especially for the late-acting loci, can cause more pronounced differences in simulation results (Figure S5 in File S1).

One could argue that an increased number of time-invariant parameters could achieve equal or better improvement. To test this, model M4 was created. In this model, parameters for the first class are fixed at the optimum values of the constant-effect class parameters obtained in M3. The remaining four classes were also replaced with constant-effect factors, keeping *p* and *n* values from M3, thus leaving γ_2_…γ_5_ to be estimated. Ten estimates with the lowest costs are presented in Table S6 in File S1. This model clearly performs worse than previous models, as can be seen from both high optimum cost (1010) and visual inspection of the best fit (Figure 3B).

### Different analysis methods have different power to detect constant- and varying-effect single nucleotide polymorphisms (SNPs)

**Figure 4:**
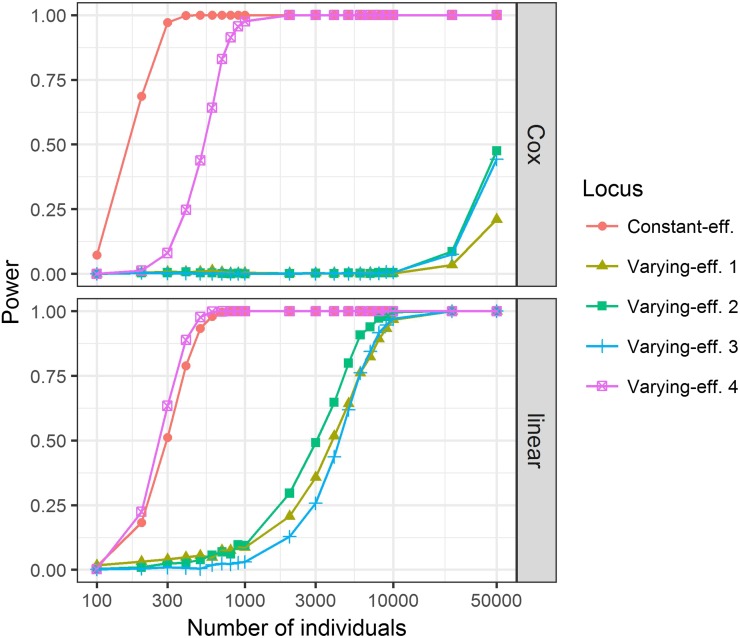
Estimates of power to detect true susceptibility loci in a genome-wide association study using Cox (above) or linear (below) regression. Five causal single nucleotide polymorphisms are modeled, using the setup and optimal parameters from M3. Numbers correspond to the alleles’ acting order during gestation, from earliest (1) to latest (4). Survival analysis had more power to detect the locus acting under proportional hazards, but loci with time-variant effects were easier to detect by linear regression. eff., effect.

Finally, we evaluated the power of different regression models to detect time-variant and time-invariant genetic effects. Genotypes were simulated using model M3 with best-fit parameters, and either linear regression or survival analysis was used to obtain p-values for each locus. If these loci were interpreted as causal SNPs in a GWAS, significance threshold for detection would be 5 × 10^−8^; Figure 4 shows how frequently the simulations produced p-values below this threshold, given various sample sizes. When the Cox model assumptions are met, as is the case for constant-effect loci, survival analysis had higher power. On the other hand, we note that varying-effect SNPs were better detected by linear regression. This can be expected under certain combinations of parameters, when a SNP dramatically changes the phenotype distribution in contradiction to the Cox model. Even though these SNPs violate the assumptions of linear regression as well, expected type I error rate is maintained in all conditions for both Cox and linear models, except when the minor allele count is 1–2 and the power to detect true effects is negligible regardless (Table S7 in File S1). Note that one of the SNPs, acting late in gestation with large effect size and MAF, shows a much higher detection power than other varying-effect loci.

## Discussion

Little is known about the genetic architecture underlying GA. Our study expands on the previous heritability studies by introducing and exploring the possibility of genetic determinants with time-variant effects. Previous studies on the same register, but using more advanced methods and different pedigrees, have estimated maternal effects at between 21 and 25% (York *et al.* 2014). However, we observe that this familial correlation does not manifest uniformly across GA, and this nonuniformity is clearly seen in plots of conditional GA distribution (Figure 1) (one possible interpretation would be to treat this distribution as a mixture of “PTD-susceptible” and “normal” populations; as the GA of one cousin increases, proportion of the “susceptible” population in the mixture decreases and the conditional distribution becomes narrower). In contrast, the same procedure applied to another phenotype, height, produces a uniform correlation pattern. Such a pattern agrees with the current understanding of height as a very polygenic trait, with thousands of SNPs contributing to the observed variation (Wood *et al.* 2014).

We attempted to replicate the GA quantile patterns by several models incorporating simulated GA loci. A model without any hereditary factors (M0), of course, does not produce correlated outcomes. Introducing one or two classes of genetic factors (models M1 and M2) produces a reasonably good fit (Figure 2). However, none of these models fully replicates the observed sloped quantiles. By incorporating mutations with time-varying effects (model M3), we were able to obtain better results, as indicated by the cost function and a better visual fit. In particular, this model replicates the observed divergence of lower quantiles seen on the left side of the correlation plots (Figure 3). This provides support for our hypothesis that factors determining GA are likely to act only in particular windows of intrauterine development.

Admittedly, model M3 could have a better fit simply because the cost function used here did not penalize for the number of new parameters introduced. Therefore, we have used several strategies to ensure comparability between the different models. First, the same parameter optimization strategy was applied to each model. Since the number of joint values over a grid grows exponentially with the number of parameters (“curse of dimensionality”), we exponentially increased the number of simulations to maintain equal sampling density. It is clear that an exhaustive search for a model with six classes of loci and three parameters per class is computationally infeasible. However, by fixing the *n* and *p* parameters at their optimum values from model M3 and enforcing time-invariant effects, we obtain model M4 with only five free parameters. High costs seen in this model (Table S6 in File S1) suggest that simply replacing the varying-effect loci in M3 with constant-effect loci will not lead to improved fit (in other words, that time-variant effects are necessary to replicate the observed quantile pattern).

Some of the design choices require additional explanation. Post-term deliveries were not investigated: all simulated survival times were capped at 300 d to replicate the clinical practice of post-term delivery induction. As our aim was an exhaustive scan of the parameter space, boundaries on effect size had to be imposed. We believe that expanding this window would not lead to more useful models: most of the top parameter combinations have effect sizes well below the preset limit of five (Tables S3 and S5 in File S1). Furthermore, higher effect sizes would impose MAFs < 0.5%. It has been suggested that these rare variants significantly contribute to the heritability of complex traits (Zhang *et al.* 2016), but such frequencies are beyond the target range of common GWAS. We also chose to use random search instead of grid search; random search is generally more efficient in high dimensionality optimization problems or when parameters have unequal importance (Bergstra and Bengio 2012). Settings for the loci with time-variant effects in M3 were picked manually, reasoning that SNPs with higher effect size (or acting earlier in gestation) should have lower risk allele frequency because of selective pressure. This relationship has been observed in real GWAS data for many traits (Park *et al.* 2011). Time of peak effect for these mutations was distributed between 230 and 258 d, *i.e.*, in the third trimester, because our models do not concern the possibility of early miscarriage. Data from other studies, although scarce, agrees with this timing. For example, the Kaplan–Meier curves generated in Harper *et al.* (2011) show that a polymorphism of IL-6 seems to act after week 33, similar to the second varying-effect mutation that we introduced in model M3. All in all, *post hoc* analyses show that the model is not particularly sensitive to these parameter values (Figure S5 in File S1).

It should be noted that the models presented here simulate a low number of causal SNPs, both in manually selected and automatically optimized parameter combinations. The reader should not make conclusions about the true number of causal factors from this: each simulated locus could be readily replaced with several factors of smaller frequency without decreasing the fit. For example, the top results for model M1 include combinations of 1 SNP with MAF 2–3% and combinations of 2–3 SNPs with MAF ∼1% (Table S3 in File S1). The same reasoning should hold if the MAF were replaced with effect size, *i.e.*, a larger number of “weaker” SNPs should be equivalent to a few “strong” ones; however, our parameter estimation consistently preferred large effect sizes for at least one of the loci. Furthermore, our power simulations show that some of the SNPs in model M3 should be easy to detect with a sample size of 1000. Previous GWA studies of PTD had even larger sample sizes, and yet did not find any significant associations, indicating that multiple rare SNPs are indeed more likely (Uzun *et al.* 2013; Zhang *et al.* 2015; Bacelis *et al.* 2016). The present models also do not account for incomplete penetrance; it would reduce the correlation caused by each SNP, requiring a larger number of loci to produce the same effect. Similar changes could be also caused by gene–gene or gene–environment interactions, which are not accounted for in this study. However, our aim was not to infer the optimal values of parameters but rather compare different models, so we believe that such omissions are acceptable.

How do these results affect design and interpretation of future PTD studies? First, we urge the obstetrics community to keep in mind that GA and PTD are time-to-event phenotypes, and to embrace survival analysis in their investigations. It can be more powerful when the proportional hazards assumption is met (Figure 4), and reanalyzing seemingly negative datasets with this approach could lead to new discoveries. Hypothesis testing under nonproportional hazards is complicated, but appropriate tools are available even for omics data (Dunkler *et al.* 2010). Time-variant genetic effects could also explain the lack of results in the GWAS of PTD so far; most of the SNPs that were used in our models are not detected at the sample sizes employed in those studies (Figure 4). It follows that researchers responsible for sample selection in such studies should consider targeting particular phenotypes or windows of GA. Finally, we welcome the use and modification of the simulation tool created in this study; the phenotype simulation is efficient, easily extendable to different functions for baseline hazards or time-variant effects, and could be used to calculate power for any statistical test of the user’s choice.

In general, while it is difficult to objectively prove that a time-variant model fits the observed data better than a model with only constant effects, this hypothesis has solid basis in biology. The presence of susceptibility windows and critical periods in human development is well-established. Up till now, this concept had not been applied to genetic studies of GA, but it would be unreasonable to believe that such a nonuniform process as human gestation could be controlled by uniformly acting determinants. In this study, we show that genetic varying-effect factors provide a simple and rational explanation of the GA heritability patterns seen in the Swedish MBR. The tools and ideas presented here should prove useful for the design of future studies, give new insights into the overall genetic architecture underlying human pregnancy, and by doing so help reduce the global health burden of PTD.

## Supplementary Material

Supplemental material is available online at www.g3journal.org/lookup/suppl/doi:10.1534/g3.116.038612/-/DC1.
