# Supplementary material for: Time-Variant Genetic Effects as a Cause for Preterm Birth: Insights from a Population of Maternal Cousins in Sweden
Source: G3 (Bethesda). 2017 Feb 28;7(4):1349–56. doi: 10.1534/g3.116.038612 (PMC5386882; doi:10.1534/g3.116.038612)
Supplement: Supplementary file 1 [file 1349file001.pdf]

SUPPLEMENTARY NOTE

1. Mathematical framework for generating survival times

Given a hazard function, under certain conditions it is possible to simulate the corresponding survival times analytically. The cumulative distribution of survival times $F(T) = 1 - S(T)$ follows a uniform distribution between 0 and 1 (Bender *et al.* 2005). Since $1 - Unif(0,1) \sim Unif(0,1)$, $S(T) = e^{-H_0(T)\, e^{\sum \boldsymbol{\beta}\mathbf{x}}} \sim Unif(0,1)$, survival times are equal to the inverse cumulative hazard function acting on a random uniformly distributed variable (here **x** represents the covariate vector, and **β** – corresponding hazard ratios):

$$T = H_0^{-1}\left(-\frac{\log(U)}{e^{\sum \boldsymbol{\beta}\mathbf{x}}}\right), \quad U \sim Unif(0,1)$$

This expression can be used to generate the survival time of any Cox model, as long as the inverse cumulative hazard function is known. For the Gompertz model used in the simulations presented here, the equivalent formula is:

$$T = \frac{1}{\alpha}\log\left(1 - \frac{\alpha \log U}{\lambda e^{\sum \boldsymbol{\beta}\mathbf{x}}}\right), \qquad U \sim Unif(0,1)$$

This formula can be used to generate survival times only when the effects of each risk factor are constant. With time-varying covariates, equivalent closed-form expressions are available only under certain conditions. Austin (Austin 2012) provides several such formulas, namely, for proportionally increasing or decreasing covariates (i.e., when $x(t) = kt$) and for covariates that flip between two states, e.g., untreated → treated → untreated. The latter formulas rely on piecewise definitions of the inverse cumulative hazard function. The main limitation in using these expressions is that covariate values

may only change at the knots[1]. For the simulation models of our study, this limitation would mean that the sensitivity periods of simulated risk factors must not overlap in time and have sharp 'on' and 'off' steps, instead of gradual increase and decrease of effect. We believe this would be a gross oversimplification of real developmental genetics. Thus, we opted to use the closed-form expression only in simulations with constant-effect genetic risk factors alone.

2. Parameter estimation

For each model, the following algorithm was used to estimate the lowest cost and parameters associated with it:

1. Draw 1000 input parameter combinations
2. Simulate the model with 1 replication for each input
3. Select 50 input combinations that result in lowest costs and determine the range of corresponding $\sum \boldsymbol{\gamma}\mathbf{pn}$ values
4. Draw new parameter combinations and exclude any inputs resulting in $\sum \boldsymbol{\gamma}\mathbf{pn}$ values outside the range defined in step 3
5. Repeat step 4 until 500 input combinations for 2-parameter models (M1, M3) or 250,000 for others (M2, M4) are obtained
6. Simulate the model with 1 replication for each input
7. Test 20 best input combinations with 20 replications

The best cost reported for each model is the best average of costs from 20 replications performed in step 7. $\sum \boldsymbol{\gamma}\mathbf{pn}$ was chosen as the predictor for metamodeling, since we observed that it showed a quadratic relationship with the model cost, resulting in a easily

[1] Some confusion may arise as the formulas elsewhere generally refer to fixed effect sizes β_x and time-variant covariates *x(t)*, while our simulations use fixed covariates G_i and time-variant effect sizes, $\gamma_i(t)$. However, as these variables always enter the model as a product $effect \times covariate$, both approaches are mathematically equivalent.

identifiable minimum. (See **Figure S2** for an example of this prediction.) It is also closely related to the mean genetic effect of each individual, as the expected value of a genotype composed from two 0/1 alleles is 2*np*.

As our models are stochastic, each simulation uses synchronized random number streams, or common random numbers (CRNs). The streams are not synchronized between replicated simulations of the same input. In this way, the observed variance of cost is reduced, and more closely reflects the 'true' variance caused by parameter differences (see Kleijnen 2015 for more details on CRNs).

Number of combinations used in step 4 was chosen to maintain comparability between 2-parameter and 4-parameter models – increasing number of tested inputs as k^m, where *m* is the number of parameters, ensures the same sampling density for all models (Hastie *et al.* 2009). The choice of *k* was assessed by bootstrap as follows. Model M1 was simulated 10,000 times with random parameters (limited by the $\sum \boldsymbol{\gamma p n}$ boundaries determined previously) to generate a distribution of costs, and 100 samples of size *k* were drawn from this distribution. We vary *k* and observe how frequently these samples contain the "true" lowest cost among the 10,000 replicates, and how far the average minimum observed per one sample of *k* simulations deviates from the "true" lowest cost **(Figure S3)**.

3. Sensitivity analysis

We performed a sensitivity analysis to evaluate how the fit of model M3 is affected by the choice of parameter values for varying-effect loci. Each of the 12 tested parameters was perturbed individually in both directions by -3 to +3 days (for *μ*) or -3 to +3 % of the best-fit value (for *p* and *γ*). Parameters for the constant-effect locus were fixed at their best-fit values. 10 iterations were performed for each parameter value. Resulting GA quantiles were averaged across the iterations to produce **Figure S5**.

LITERATURE CITED

Austin, P. C., 2012 Generating survival times to simulate Cox proportional hazards models with time-varying covariates. Stat. Med. 31: 3946–3958.

Bender, R., T. Augustin, and M. Blettner, 2005 Generating survival times to simulate Cox proportional hazards models. Stat. Med. 24: 1713–1723.

Hastie, T., R. Tibshirani, and J. Friedman, 2009 *The Elements of Statistical Learning*. Springer New York, New York, NY.

Kleijnen, J. P. C., 2015 *Design and Analysis of Simulation Experiments*. Springer.

SUPPLEMENTARY FIGURES AND TABLES

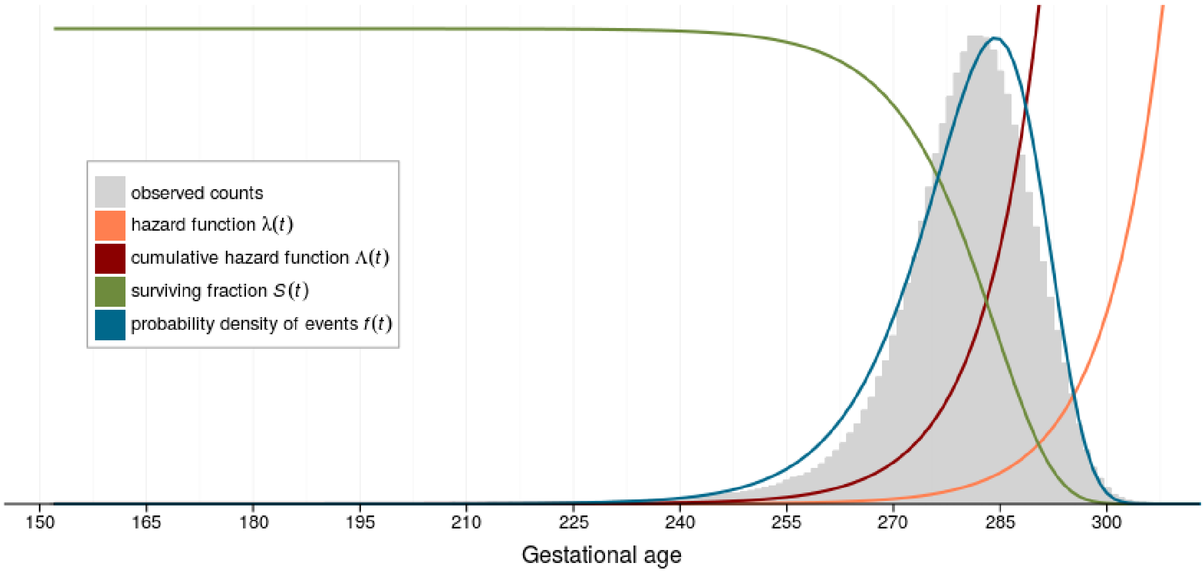

Figure S1. Dynamics of the baseline survival model. Grey histogram – actual gestational age data, observed in the entire Swedish Medical Birth Register (>1.3 million observations). Corresponding density of gestational ages, simulated using the baseline Gompertz model with optimal parameters, is shown in blue. Remaining curves show the simulated "hazard" to be born, corresponding cumulative "hazard", and proportion of fetuses remaining *in utero* at each day of gestation. As all these functions are measured in different units and were rescaled for presentation purposes, *y*-axis is shown without scale.

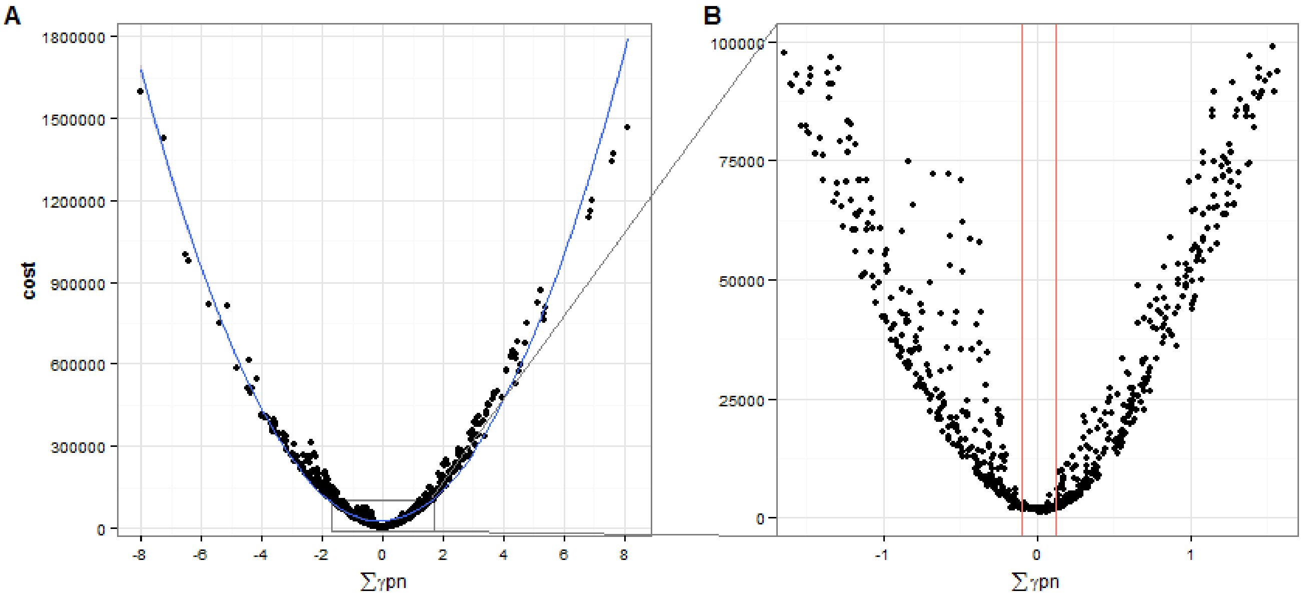

Figure S2. A – Metamodeling of cost as a quadratic function of predictor $\sum \boldsymbol{\gamma}\mathbf{pn}$, model M1. Best-fit parabola $\boldsymbol{y = 22521x^2 + 1414x + 37126}$ is shown in blue. Since a clear minimum is observed, metamodeling allows to constrain the parameter space to a certain range of predictor values. Highlighted section is shown in detail in B. Red lines indicate the minimum and maximum predictor values (-0.098 and 0.1277, respectively) observed for top 50 results. Only parameter combinations having $\sum \boldsymbol{\gamma}\mathbf{pn}$ within these boundaries will be tested when searching for the best fit of this model. Note also that a clear lower limit of cost exists for each predictor value, while the upper limit is less defined.

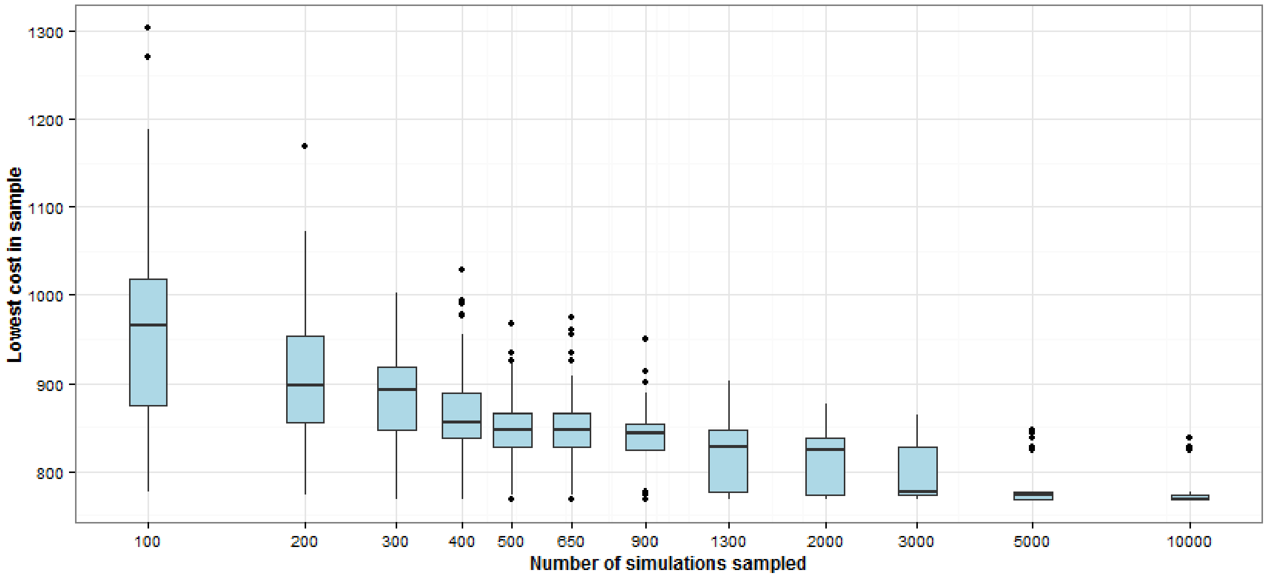

Figure S3. Choice of 500 simulations per model was assessed by bootstrapping samples of various size from 10,000 simulations of M1. Boxes show the median and interquartile range of minimum cost observed in each sample. Using <3000 simulations, most of the samples did not contain the “true” minimum observed among 10,000 simulations. However, the average difference between the “true” minimum and the lowest cost in samples of 500 simulations was only about 80 units, therefore, to save computing time, this number was chosen for further use.

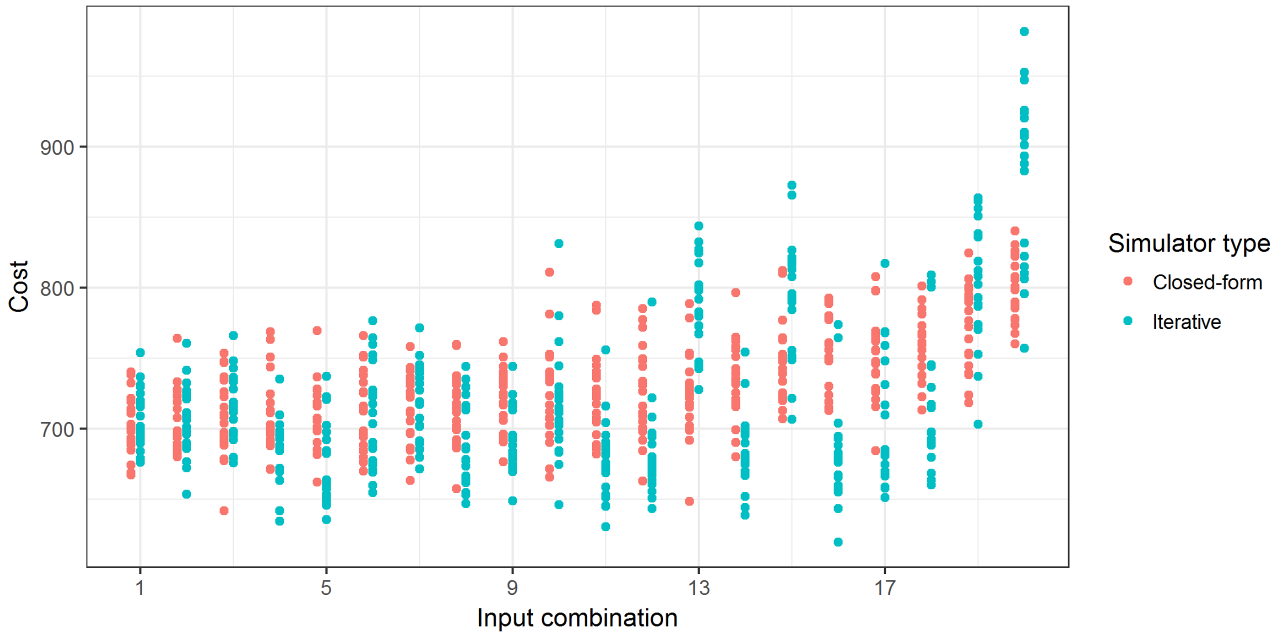

Figure S4. Comparison of different simulator types. X axis represents 20 best combinations of inputs for model M2, determined by iterative simulator. For each input combination, 20 replications were simulated with both simulators (dots).

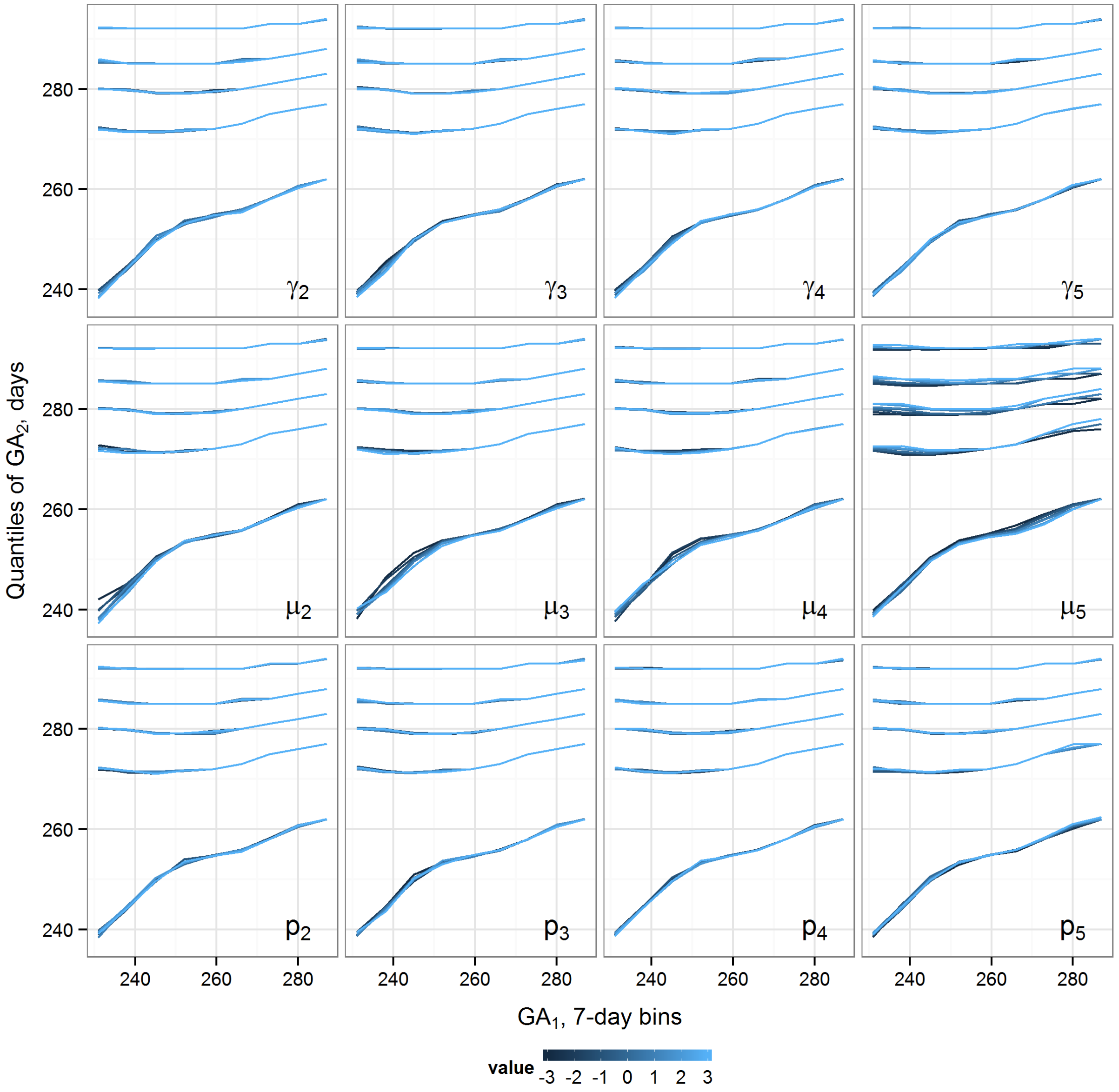

Figure S5. Sensitivity analysis of model M3. Each panel shows simulation results produced by perturbing one parameter (indicated in the corner). Perturbations range from -3 to +3 % of the initial value (for *γ* and *p*), or from -3 to 3 days (for *μ*). High overlap between curves, as seen in *γ* and *p* parameter plots, indicates that the results are relatively insensitive to the choice of these parameter values. In contrast, timing of the late-acting locus (μ_5) can have a stronger effect on the simulation results.

Term	β	SE(β)	p-value
(Intercept)	308.4	3.1	$<10^{-6}$
Parity 2	0.215	0.02	$<10^{-6}$
3	0.468	0.03	$<10^{-6}$
≥4	-0.196	0.04	9.99×10^{-6}
Gender (female)	-0.208	0.09	$<10^{-6}$
Maternal height, per 1 cm	0.114	0.002	$<10^{-6}$
Birth year, per 1 year	-0.0249	0.002	$<10^{-6}$
Maternal weight, per 1 kg	0.0337	0.001	$<10^{-6}$
Marital status (other than married)	-0.0326	0.04	0.37
Maternal age ≤20 years	-0.328	0.05	$<10^{-6}$
>25, ≤30 years	0.294	0.05	$<10^{-6}$
>30, ≤35 years	0.530	0.05	$<10^{-6}$
>35 years	0.234	0.06	$<10^{-6}$
Mother born not in Sweden	-0.293	0.02	$<10^{-6}$
Smoking 1-9 cigarettes/day	-0.852	0.04	$<10^{-6}$
≥10 cigarettes/day	-1.837	0.05	$<10^{-6}$

Table S1. Coefficients, standard errors and p-values obtained from multivariable linear regression model, used to adjust gestational age. Coefficients for categorical variables represent differences in gestational age in days, compared to a corresponding reference group of primiparous, married, Swedish-born, non-smoking mothers between 21-25 years, having a male child.

Distribution function	Best fit parameters	AIC score of best fit
Exponential	*rate*=7.712×10^{-3}	15,402,346
Weibull	*shape*=15.88, *scale*=1.669×10^{-34}	9,680,240
Gompertz	*shape*=0.1212, *rate*=1.026×10^{-8}	9,640,465

Table S2. Parameters and AIC scores obtained from fitting three survival distribution to the gestational ages observed in Swedish MBR. Values are presented following the parameterization used in 'flexsurv' R package.

γ	n	p	mean cost	SD of cost
2.21	1	0.019	**796**	51.2
2.01	3	0.00795	**819**	45.5
2.22	2	0.0117	**824**	77.0
3.36	1	0.00907	**840**	56.5
2.35	1	0.0262	**935**	29.9
2.69	2	0.00964	**946**	47.9
2.25	1	0.0292	**960**	37.5
2.48	3	0.00856	**998**	20.4
2.09	1	0.0375	**1030**	26.4
2.60	3	0.00804	**1030**	23.2

Table S3. 10 input parameter combinations resulting in the lowest costs for model M1. Mean and SD represent the average and standard deviation of costs obtained from 20 replications.

γ_1	n_1	p_1	γ_2	p_2	mean cost	SD of cost
**2.56**	2	0.00857	-0.326	0.043	**655**	19.9
**-0.36**	1	0.0242	2.79	0.0133	**671**	17.6
**-1.42**	1	0.00398	2.57	0.0147	**671**	23.6
**-0.101**	1	0.218	2.72	0.0163	**685**	19.8
**-0.314**	1	0.0734	2.50	0.0189	**688**	24.3
**2.80**	3	0.00399	0.808	0.00273	**688**	29.6
**-0.547**	1	0.0346	2.56	0.0182	**695**	32.5
**0.15**	3	0.00878	2.60	0.0142	**699**	48.2
**-0.058**	2	0.286	2.41	0.0239	**702**	16.8
**-0.075**	2	0.197	2.42	0.0205	**706**	22.4

Table S4. 10 input parameter combinations resulting in the lowest costs for model M2. Mean and SD represent the average and standard deviation of costs obtained from 20 replications.

γ	n	p	mean cost	SD of cost
0.851	1	0.211	**338**	30.0
0.817	1	0.225	**348**	44.6
0.892	2	0.101	**351**	36.2
0.82	1	0.225	**355**	34.9
0.763	1	0.252	**362**	36.5
0.79	2	0.109	**366**	28.9
0.97	2	0.0932	**369**	47.9
0.952	3	0.0632	**373**	34.2
0.746	4	0.0638	**373**	38.8
0.655	2	0.136	**374**	40.7

Table S5. 10 input parameter combinations resulting in the lowest costs for model M3. Mean and SD represent the average and standard deviation of costs obtained from 20 replications.

γ_2	γ_3	γ_4	γ_5	mean cost	SD of cost
2.87	-0.237	-0.208	-0.519	**1010**	19.6
3.02	-0.495	-0.417	-0.386	**1020**	23.0
2.82	0.0863	-0.319	-0.45	**1020**	31.0
2.8	-0.253	-0.844	-0.436	**1020**	29.9
2.52	-0.223	-0.036	-0.487	**1030**	28.8
2.65	-0.707	-0.109	-0.488	**1030**	38.2
2.92	-1.63	-0.0547	-0.462	**1040**	27.5
2.91	-2.58	-0.571	-0.404	**1070**	23.5
2.39	-0.409	-0.931	-0.397	**1080**	37.2
2.5	-1.26	0.213	-0.501	**1090**	29.2

Table S6. 10 input parameter combinations resulting in the lowest costs for model M4. Mean and SD represent the average and standard deviation of costs obtained from 20 replications.

Model	Sample size	T1E rate with MAF=0.01	T1E rate with MAF=0.3
**Linear**	100	0.055	0.049
	200	0.065	0.052
	500	0.053	0.059
	700	0.049	0.043
	1000	0.052	0.049
	5000	0.050	0.062
	10000	0.054	0.048
	50000	0.056	0.054
**Cox**	100	0.097	0.057
	200	0.080	0.053
	500	0.062	0.058
	700	0.051	0.049
	1000	0.059	0.055
	5000	0.044	0.053
	10000	0.064	0.053
	50000	0.053	0.039

Table S7. Type I error rate calculations. Two non-causal SNPs were tested by linear or Cox regression models, while the phenotype was simulated by model M3 with best-fit parameter values. Reported numbers are fractions of p-values below 0.05. The error rate holds at the expected value around 0.04-0.06 for most scenarios, except when the Cox model is applied to loci with 1-2 counts of the minor allele.
